# 

**Published:** 1896-09

**Authors:** 


### TABLE OF CONTENTS.

ORIGINAL COMMUNICATIONS. PAGE

Nasal Obstructions which result in Mouth-Breathing, with Special Reference to Adenoid Vegetations. By GEORGE L. RICHARDS, M.D., Boston, Mass. . . . . . 549

Dental Uses for Röntgen's Discovery.—Support for Tube. By WILLIAM ROLLINS, Boston, Mass. . . . . . 559

An Apparent Case of Salivation. By C. W. STAINTON, D.D.S., Buffalo, N. Y. . . . . . 561

Dentistry, Practical and Theoretical. By WILLIAM SHAW TWILLEY, Baltimore, Md. . . . . . 563

ABSTRACTS AND TRANSLATIONS.

Salubrol, a Substitute for Iodoform. By PAUL DE TERRA, Dentist, Zürich, Switzerland . . . . . 566

Eucaine Hydrochlorate, a New Local Anæsthetic. By DR. GAETANO VINCI, Messina, Sicily . . . . . 572

Eucaine Hydrochlorate. By H. KIESEL, Dentist, Berlin, Germany . . . 576

REPORTS OF SOCIETY MEETINGS.

The New York Institute of Stomatology . . . . . 580

Central Dental Association of Northern New Jersey . . . . . 599

EDITORIAL.

Saratoga, 1896 . . . . . 617

